# Synthesis, characterization, and debromination reactivity of cellulose-stabilized Pd/Fe nanoparticles for 2,2',4,4'-tretrabromodiphenyl ether

**DOI:** 10.1371/journal.pone.0174589

**Published:** 2017-03-29

**Authors:** Guofu Huang, Mianmian Wang, Yongyou Hu, Sihao Lv, Changfang Li

**Affiliations:** 1 School of Environment and Energy, South China University of Technology, the Key Lab of Pollution Control and Ecosystem Restoration in Industry Clusters, Ministry of Education, Guangzhou, PR China; 2 State Key Laboratory of Pulp and Paper Engineering, South China University of Technology, Guangzhou, PR China; 3 School of Environment and Civil Engineering, Dongguan University of Technology, Dongguan, PR China; 4 Dongguan Cleaner Production Center, Dongguan, PR China; Brandeis University, UNITED STATES

## Abstract

In this study, two kinds of cellulose derivatives (polyanionic cellulose (PAC) and hydroxypropylmethyl cellulose (HPMC)) were selected as stabilizers of Pd/Fe nanoparticles (NPs) to investigate their influences on the debromination performances of 2,2',4,4'-tretrabromodiphenyl ether (BDE47). Field emission scanning electron microscope (FE-SEM) images revealed that the cellulose-stabilized Pd/Fe NPs were smaller and more uniform than the bare-Pd/Fe NPs. X-ray diffractometer (XRD) and X-ray photoelectron spectroscopy (XPS) results suggested that cellulose coatings found on Pd/Fe NPs surfaces featured some antioxidation abilities, which followed the order of HPMC < PAC. Sedimentation tests demonstrated that the stabilizing power of PAC for Pd/Fe NPs was higher than that of HPMC. Fourier transfer infrared spectrometer (FTIR) results indicated that PAC molecules were bound to the Pd/Fe NPs surfaces by polar covalent bonds and hydrogen bonds, while HPMC molecules interacted with the nanoparticles by hydrogen bonds. Batch debromination test for BDE47 demonstrated that the catalytic debromination rate with cellulose-stabilized Pd/Fe NPs was higher than that with bare-Pd/Fe NPs during reaction period of 15 min. Overall, this study indicated that both celluloses are beneficial to forming smaller, more regular, stable and antioxidative Pd/Fe NPs, leading to higher debromination reactivity for BDE47 compared with the bare-Pd/Fe NPs. Therefore Pd/Fe NPs can be utilized as a promising remediation technology for the contaminated groundwater and soils.

## Introduction

In the past two decades, zero-valent iron (ZVI) and nanoscale zero-valent iron (NZVI) have attracted great interest for their promising applications in the remediation of groundwater and soils contaminated with heavy metals [1–3], nitrate [4, 5], halogenated hydrocarbons [6, 7], phenolic compounds [8], polychlorinated biphenyls (PCBs) [9, 10], and polybrominated diphenyl ethers (PBDEs) [11–14]. Compared to the conventional ZVI particles, NZVI can offer an advantage of their large specific surfaces, this can provide more active sites for the surface-mediated reaction, leading to faster degradation rate of contamination. In addition, NZVI are more flexibly delivered into the polluted soils, groundwater, sediments, and aquifers for in situ remediation since their nano-scale particle size [15–17]. However, due to the extremely attractive interparticles van der Waals and magnetic forces, NZVI tended to aggregate rapidly in contaminated sites to form micron or even millimeter-scale aggregates, therefore limiting their mobility and decreasing their reactivity [18]. In addition, the highly reactive NPs may react with the surrounding media, such as dissolved oxygen, water and other impurities, reducing their reactivity [19, 20].

A variety of stabilizers have been used to improve the dispersity and stability of Fe^0^-based NPs and protect them from aggregating. Synthetic polymers and natural biopolymers (such as carboxymethyl cellulose (CMC) [21–24], polyacrylic acid (PAA) [25, 26], polyvinylpyrrolidone (PVP) [27, 28], polyaspartate (PAP) [29], and starch[30] have been proved to be promising stabilizers due to their high water solubility, low cost, and environmental compatibility. Stabilized ZVI NPs with these polymers during their synthesis have been shown to overcome attractive van der Waals forces and magnetic forces by the electrostatic repulsion and/or steric hindrance. This can inhibit their aggregation and improve their stability, transport, and reactivity. Recently, CMC (a chemical cellulose derivative) bearing carboxymethyl groups and hydroxyl groups on the molecule backbone has been successfully used to stabilize ZVI NPs [21–24]. He et al. [22] employed CMC as a stabilizer during ZVI NPs synthesis, which resulted in much smaller ZVI NPs when compared to bare-ZVI NPs. Sakulchaicharoen et al. [28] compared the degradation rate of trichloroethylene (TCE) by CMC, PVP, and guar gum stabilized Pd/Fe NPs, and found that much smaller CMC-Pd/Fe NPs had higher reactivity.

Polyanionic cellulose (PAC) is water-soluble anionic cellulose ether, which is synthesized using an alkali-catalyzed method. Compared to CMC, PAC has higher purity and degree of substitution [31]. Besides, PAC has intrinsically outstanding characteristics, including superior resistance to heat, excellent tolerance to salt, and strong antibacterial activity [32]. To the best of our knowledge, there are no related reports using PAC as a nanoparticle stabilizer. In addition, hydroxypropylmethyl cellulose (HPMC) bearing a large amount of hydroxyl groups is also an important chemical cellulose derivative [33, 34]. Tiwari et al. [33] found that HPMC was much more effective at nucleating and stabilizing colloidal ZnS nanoparticles in aqueous suspensions compared with poly(vinyl alcohol) (PVA) and starch. However, up to date, the available data about stabilization mechanism and enhanced degradation reactivity on contaminations by HPMC stabilized Pd/Fe NPs is still scarce.

In order to develop environment-friendly and low-cost stabilizer to improve the stability and reactivity of iron-based NPs, we selected two kinds of cellulose derivatives (PAC, and HPMC) as potential stabilizers for Pd/Fe NPs. In this study, 2,2',4,4'-tretrabromodiphenyl ether (BDE47), a class of typical brominated flame retardants, is adopted as a target contamination to test the reactivity of Pd/Fe NPs. A nonionic surfactant Brij35 as a solubilizer is added into aqueous solution due to the high hydrophobicity of BDE47 according to the related study [35].

The overall objective of this study is as follows: (i) to investigate the effect of stabilizers (PAC and HPMC) on crystal structure, morphology, particle size, chemical composition and suspension stability of Pd/Fe NPs; (ii) to explore the possible stabilization mechanism of these celluloses; (iii) to evaluate the debromination reactivity of cellulose-stabilized Pd/Fe NPs for BDE47.

## Materials and methods

### Chemicals

Ferrous sulfate heptahydrate (FeSO_4_·7H_2_O, AR), sodium borohydride (NaBH_4_, AR), NaPdCl_4_·3H_2_O (AR), and Brij 35 (nonionic surfactant, C_12_H_25_(OCH_2_CH_2_)_23_OH, AR) were purchased from Macklin Biochemical CO. Ltd. (Shanghai, China). BDE47 (99.5%) was obtained from Chem. Service Inc. (West Chester, USA). Hydroxypropylmethyl cellulose (HPMC, USP2910, 2% viscosity: 50 mPa·s) was purchased from Aladdin (Shanghai, China). Polyanionic cellulose (PAC, AR) was obtained from Rite Chemical CO. Ltd. (Foshan, China). Methanol was purchased from Merck Co., Ltd. (Shanghai, China). In this study, deoxygenated deionized water was used in all experiments.

### Preparation of nanoparticles

The modified Pd/Fe NPs were synthesized in a 250 mL anaerobic four-necked flask with mechanical stirring under N_2_ atmosphere. The synthesis steps were as follows according to previous literatures [21]: Firstly, FeSO_4_·7H_2_O was added to 90 mL of cellulose (PAC or HPMC) solutions, yielding a desired concentration of Fe^2+^ and celluloses. The mixture was then stirred for 20 min at 300 rpm to ensure the formation of Fe^2+^-cellulose complex. Secondly, 5 mL of NaBH_4_ solution was added dropwise to the Fe^2+^-cellulose complex solution at a BH_4_^-^/Fe^2+^ molar ratio of 2.0. NPs were formed and the reaction could be depicted by the following reaction equation:

$${{Fe(H_{2}O)}_{6}}^{2+}+2{BH}^{-}\to {Fe}^{0}+2B{\left(OH\right)}_{3}+7H_{2}↑$$ (1)

When hydrogen evolution ceased, 5 mL of K_2_PdCl_6_ aqueous solution was added to Fe^0^-cellulose complex solution after 15 min. Pd^2+^was then reduced by Fe^0^NPs, resulting in cellulose-stabilized Fe/Pd bimetallic NPs according to the following equation:

$$Pd{Cl}_{6}^{2-}+2{Fe}^{0}\to 2{Fe}^{2+}+{Pd}^{0}+6{Cl}^{-}$$ (2)

In order to get rid of the excess chemicals, the resulted black suspension was centrifuged at a high speed (10000 r/min) for 15 min. And then the supernatant was removed, the black precipitates were rinsed successively with deionized water and absolute ethanol for 2 times in an anaerobic atmosphere. All the NPs were dried at 60°C in a vacuum drying oven. For comparison, bare-Pd/Fe NPs were also synthesized according to the aforementioned method just without addition of cellulose.

For simplicity, Pd/Fe nanoparticles stabilized by PAC and HPMC are respectively denoted as PAC-Pd/Fe and HPMC-Pd/Fe NPs throughout the manuscript, Pd/Fe nanoparticles which are not modified by cellulose are denoted as bare-Pd/Fe NPs.

### Characterizations

The morphological characteristics of bare-Pd/Fe, HPMC-Pd/Fe, and PAC-Pd/Fe NPs were analyzed by a field emission scanning electron microscope (FE-SEM, Merlin, Zeiss Ltd., Germany) at an operating voltage of 5 kV. These samples were sprayed with a thin electric conductive gold film before being scanned for all cases. Brunnaer–Emmett–Teller (BET) specific surface areas of the bare and cellulose-modified Pd/Fe NPs were measured using an ASAP2010 surface analyzer (Micromeritics Instrument, USA) and with a N_2_ adsorption method. The crystal structure was characterized by a X-ray diffractometer (XRD, Rigaku, Co., Japan) with a high-power Cu-Kα radioactive source (λ = 1.5418 Å) at an accelerating voltage of 45 kV and emission current of 40 mA. All samples were scanned from 10 to 100 2*θ* at a scanning rate of 3° 2*θ* per minute. X-ray photoelectron spectroscopy (XPS) analysis for bare and modified nanoparticles was performed by an ESCALAB 250 instrument (Thermo-VG Scientific Co., USA), with a Mg Kα radiation source (photonelectron energy 1486.6 eV) at a power of 300 W. The binding energies of the photoelectron were calibrated by the aliphatic adventitious hydrocarbon C1s peak at 284.6 eV. Fourier transfer infrared spectrometer (FITR, VERTEX 70, Bruker, GER) was employed to investigate the surface chemical structure and composition of cellulose modified nanoparticles. The dried samples were mixed with KBr and pressed into pellets consisting of 1.5% (w/w) of the nanoparticles. All spectra were collected with a resolution of 4 cm^−1^ in the range of 4000–600 cm^−1^. Zeta (ζ) potentials of Pd/Fe NPs suspensions were measured using a Zeta Potential Analyzer (Zetasizer Nano ZS, Malvern, England) at room temperature. Solution pH value was adjusted to 7.0 by adding 0.5 M H_2_SO_4_ or 1.0 M NaOH. Averaged data were obtained from three independent measurements. The stability of all Pd/Fe NPs suspensions was evaluated by monitoring the change in absorbance at λ = 508 nm over time under static conditions. A UV–vis spectrophotometer (HACH, DR5000, USA) was used for absorbance measurements of the suspension in sealed cuvettes to prevent air oxidation. The pH values of all suspensions containing 0.4 g L^-1^Pd/Fe NPs were adjusted to 7.0.

### Degradation of BDE47

The experiments for degradation of BDE47 were carried out in 250 mL four-necked flask covered with Teflon-lined caps at room temperature. The BDE47 stock solution (1000 mg L^-1^) was prepared using methanol as solvent and stored in a refrigerator at 4°C. 0.04 g of freshly synthesized cellulose-stabilized Pd/Fe NPs and 99.9 mL Brij35 surfactant solution (1 g L^-1^) were added to the flask. BDE47 degradation was initiated by spiking 0.1 mL of BDE47 stock solution into the flask to obtain a desired initial BDE47 concentration (1 mg L^-1^). The flask was stirred at 300 rpm. At selected time, 2 mL aqueous samples were withdrawn from the reactor using a 5 mL glass syringe and then filtered through 0.45 μm cellulose acetate membrane (CA) filters for future analysis. Control experiments without addition of the nanoparticles were conducted in parallel at the same time. All samples were prepared in triplicate and the averaged data were reported.

The pseudo-first-order kinetic model is used to fit the data of BDE47 debromination reaction, which could be represented in the following equations:

$$-\frac{dC}{dt}=k_{obs}C=k_{SA}a_{s}\rho_{m}C$$ (3)

$$t_{1/2}=-\frac{\ln\;0.5}{k_{obs}}$$ (4)

where *C* is the BDE47 concentration (mgL^-1^) in the aqueous solution at time of *t* (min), *k*_*obs*_ is the observed pseudo-first-order kinetic rate constant (min^-1^), *k*_*SA*_ is the surface-area-based rate constant (L min^-1^ m^-2^), *a*_*s*_ is the BET specific surface area of the NPs (m^2^ g^-1^), *ρ*_*m*_ is the mass concentration of the NPs (g L^-1^), and *t*_*1/2*_is the half-life period of BDE47 debromination (min).

### Analytical methods

The concentration of BDE47 was analyzed at 226 nm with a high performance liquid chromatography (HPLC, Waters, e2695, USA) equipped with a 2998 Photodiode Array Detector. A reversed-phase column (SunFire C_18_, 5 μm, 4.6×250 mm) was used and the column temperature was maintained at 30°C. The HPLC was run with a mobile phase of methanol/H_2_O (93/7, V/V) at a flow rate of 1.0 mL min^−1^. The injected sample volume was 20 μL. The samples were only filtered with 0.45 μm cellulose acetate membrane (CA) filters without further processing.

## Results and discussion

### Morphology, particle size distribution and BET specific surface area

**Fig 1:**
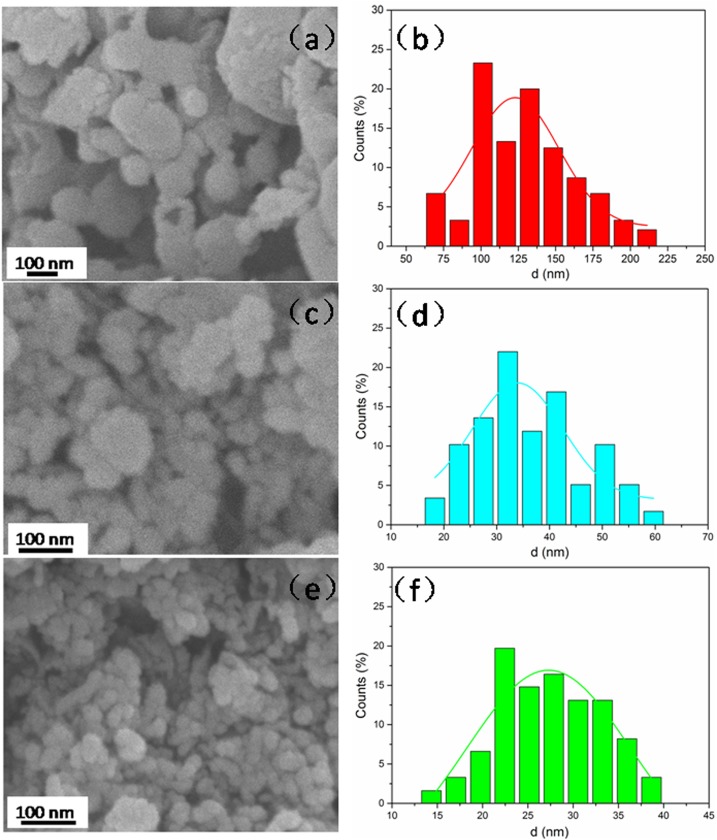
FE-SEM images of (a) bare-Pd/Fe, (c) HPMC-Pd/Fe, and (e) PAC-Pd/Fe NPs; (b), (d), and (f) are the particle size distribution histograms of NPs corresponding to (a), (c), and (e). Fe loading is 0.4 g L-^1^ with Pd 0.3 wt% of Fe, cellulose is 1 g L^-1^.

The morphologies and particle size distributions of bare-Pd/Fe and cellulose-stabilized Pd/Fe NPs are shown in Fig 1. Fig 1(A) shows that bare-Pd/Fe NPs with large particle size and irregular shape aggregated severely. Almost no regular particles could be easily distinguished from these agglomerated clusters because of the severe magnetic forces and van der Waals interactions between them. In contrast, both HPMC-Pd/Fe (Fig 1(C)) and PAC-Pd/Fe (Fig 1(E)) NPs with spherical shape are far smaller in size and more uniform in shape when compared with bare-Pd/Fe NPs. This result indicates that these two kinds of celluloses play key roles in obtaining smaller and more uniform NPs. Besides, PAC-Pd/Fe NPs have smoother surfaces and smaller sizes compared with HPMC-Pd/Fe NPs, implying that PAC is more beneficial to synthesize regular and small NPs. It might be because PAC can be bound to the nanoparticle surface much better.

Fig 1(B), 1(D) and 1(F) further reveal the particle size distributions of bare-Pd/Fe, HPMC-Pd/Fe, and PAC-Pd/Fe NPs, respectively. The sizes were measured by the same number of NPs using the Nano Measurer 1.2 software. The mean sizes of bare-Pd/Fe, HPMC-Pd/Fe, and PAC-Pd/Fe NPs are estimated to be 132.5±18.9 nm, 35±8.1 nm, and 27±5.2 nm, respectively, which indicates that all cellulose-stabilized Pd/Fe NPs are smaller than bare-Pd/Fe NPs. It is in good accordance with the previous researches. He et al. and Sakulchaicharoen et al. considered that polymer could accelerate the growth of the attached particles (nucleation) by serving as a network in the synthesis process of iron-based NPs, which led to formation of a great deal of smaller particles [22, 28]. When nucleation was completed, the polymer molecules can be attached to the particle surface, and prevent further growth of the NPs due to electrostatic repulsion and steric hindrance [22]. Similar conclusion appears to be plausible for all cellulose-stabilized Pd/Fe NPs.

**Fig 2:**
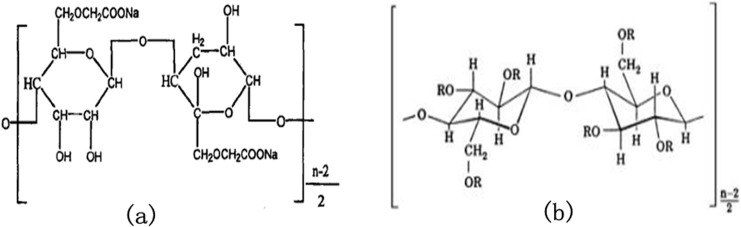
Molecular structures of PAC (a) and HPMC (b). The substituent R of HPMC molecular represents either a–CH_3_, or a–CH_2_CH(CH_3_)OH, or a hydrogen atom.

Differences in the estimated mean particle sizes between HPMC-Pd/Fe and PAC-Pd/Fe NPs can be explicated through different molecular structures of these two celluloses. As shown in Fig 2, PAC molecule bears carboxylate (-COO^-^) and hydroxyl (OH^-^) functional groups, while HPMC molecule only bears hydroxyl functional groups. Besides, the molecular weight of PAC (700 K) is greater than HPMC (86 K), seen in Table 1. There are three reasons that could cause the differences in sizes between cellulose-stabilized Pd/Fe NPs. First, PAC could exert stronger netting effects on Fe^2+^ ions because of its greater molecular weight when compared with HPMC. Therefore, it could much effectively improve the Fe cluster nucleation rate at the early stage of iron reduction. He et al [22]. proposed that a faster cluster nucleation rate would favor the production of more and smaller NPs since there was no sufficient time for cluster agglomeration during the process. This could be a major reason for the production of large amounts of smaller NPs in the present of PAC. Meanwhile, the PAC-Fe^2+^ complexes (i.e., precursors of Fe NPs) are quickly formed through the electrostatic interactions between Fe^2+^ ions and carboxylate groups (-COO^-^) on PAC molecules. But in the case of HPMC, the Fe^2+^ ions probably form weak bonds with the lone pair electrons at the oxygen atoms on the HPMC hydroxyl groups. Such bonding could also occur on PAC-Fe^2+^ complex since PAC molecules also bear hydroxyl groups. Therefore, the interaction between PAC and Fe^2+^ is stronger than that between HPMC and Fe^2+^ because the carboxylate group of PAC bears a net negative charge compared to the lone pair electron at the HPMC hydroxyl group. Sakulchaicharoen et al. [28] proposed that the stronger the interaction between polymer and Fe^2+^ is, the faster the nucleation rate of Fe cluster is. As the interaction between PAC and Fe^2+^ is stronger than that between HPMC and Fe^2+^, this results in improved Fe cluster nucleation rate in PAC mixture once sodium borohydride was added. This could be another reason for the production of large amounts of smaller NPs in the present of PAC by charge effect of celluloses. Besides, once the NPs are formed, PAC molecules with greater molecular weight and net negative charge are bound to the particle surface, and would prevent further growth of NPs by stronger steric hindrance and electrostatic repulsion. But, in the case of HPMC (a non-ionic cellulose) with a smaller molecular weight, it can only provide weaker steric hindrance to restrict particles growth. This may be the third reason that HPMC is not as effective as that of PAC to stabilize Pd/Fe NPs.

**Table 1:** Molecular weight, *pKa*, and degree of substitution (D.S.) of PAC and HPMC.

Stabilizer	Molecular weight	*pKa*	D.S.
PAC	700K	4.3	0.9
HPMC	86K	-	a

a, The contents of methylol groups and hydroxypropyl groups on HPMC are 27.5% and 2.0%, respectively.

The specific surface areas of bare and stabilized Pd/Fe NPs were measured with the BET method. The values of specific surface areas for bare-Pd/Fe, HPMC-Pd/Fe, and PAC-Pd/Fe NPs are 32.3±1.8, 43.8±3.2, and 58.6±4.5 m^2^ g^-1^, respectively. It is generally considered that the specific surface area of the particles could be increased as their size decreased [26], thus the result here has indicated clearly that the particle sizes of cellulose-stabilized Pd/Fe NPs are smaller than bare-Pd/Fe NPs. This result is in good accordance with the results of SEM.

### XRD and XPS analysis of NPs

**Fig 3:**
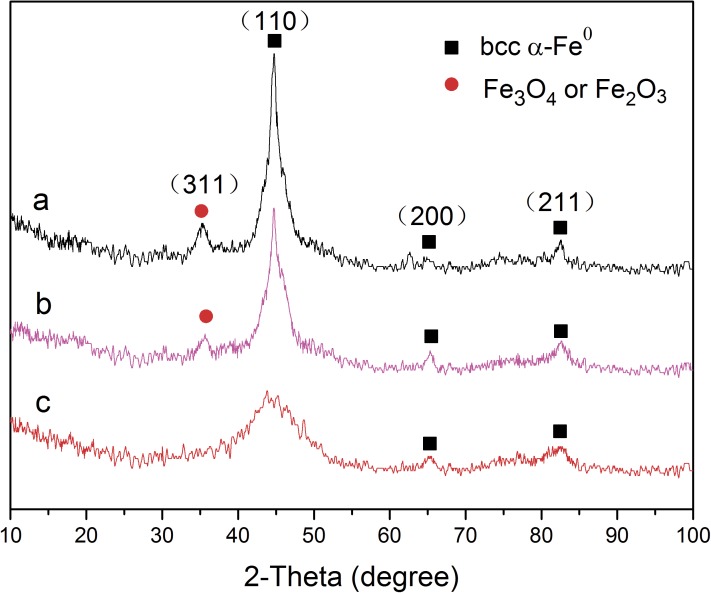
XRD images of (a) bare-Pd/Fe, (b) HPMC-Pd/Fe, and (c) PAC- Pd/Fe NPs. Fe loading is 0.4 g L^-1^ with Pd 0.3 wt% of Fe, cellulose is 1 g L^-1^.

XRD is used to examine the chemical composition and crystal structure of bare and stabilized Pd/Fe NPs. As shown in Fig 3, no characteristic peak belonging to Pd is observed due to the extremely low Pd content (0.2 wt%) in all Pd/Fe nanoparticle samples. Three characteristic diffraction peaks at 2*θ* of 44.80°, 65.32°, and 82.60° correspond to the 110, 200, and 211 diffraction peaks of iron, respectively. They are clearly observed in bare and stabilized Pd/Fe NPs, which suggests the existence of bccα-Fe^0^ in all Pd/Fe NPs samples [25]. For bare-Pd/Fe NPs, the 311 diffraction of iron oxide (peak at 35.32° for*γ*-Fe_2_O_3_ or Fe_3_O_4_) [36, 37] is found distinctly in the XRD pattern, implying a certain amount of bare-Pd/Fe NPs were oxidized in the synthesis process. There is weaker oxidation peak found in XRD patterns of HEMC-Pd/Fe NPs compared with bare-Pd/Fe NPs. No oxidation peak appears at 2*θ* = 35.62° in the XRD image of PAC-Pd/Fe NPs. The results can be explained by which the outer coating of celluloses can protect the stabilized NPs from being oxidized in the synthesis process when compared with bare-Pd/Fe NPs. Similar results were reported by Lin et al [25], where the poly acrylic acid (PAA250K) could protect the surroundingFe^0^ from further corrosion and prevent the bimetal from being oxidized in the air.

**Fig 4:**
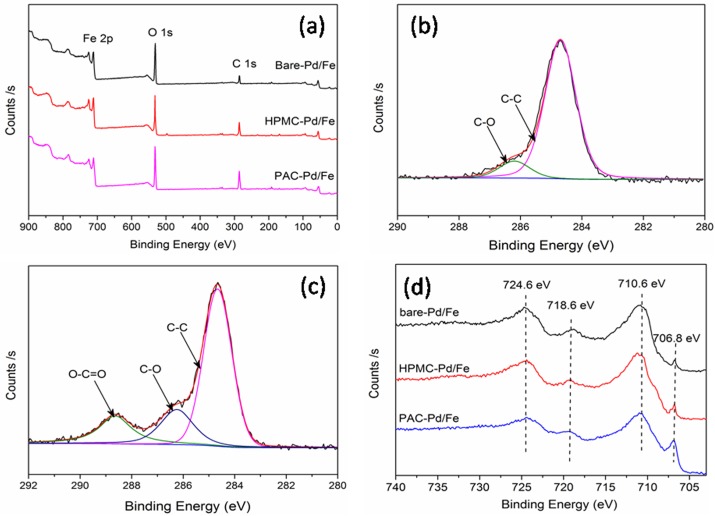
XPS images of bare and cellulose-modified Pd/Fe NPs: (a) full-range XPS spectra, (b) C1s of bare-Pd/Fe NPs, (c) C1s of PAC-Pd/Fe NPs, (d) Fe 2p spectra. Fe loading is 0.4 g L^-1^ with Pd 0.3 wt% of Fe, cellulose is 1 g L^-1^.

XPS is introduced to investigate the surface chemical compositions of bare and cellulose-stabilized Pd/Fe NPs. The whole region scan survey reveals that Fe, O, and C are the principal elements of all NPs surfaces. The C1s core-level spectrum of bare-Pd/Fe NPs (Fig 4(B)) is fitted to two peaks at 284.7 and 286.2 eV, corresponding to the C-C and C-O species [38], respectively. These carbon peaks may come from water, ethanol, or/and carbon dioxide contamination in the process of sample preparation [39]. After Pd/Fe NPs were stabilized with PAC, the noticeable variation appears in C1s core-level spectrum (Fig 4(C)). The C1s spectrum of Pd/Fe NPs was decomposed into three curves with peaks at 284.7, 286.4, and 288.8 eV, which presents C-C, C-O, and O = C-O species [38], respectively. This result indicates PAC molecule might be adsorbed or bound to the surface of PAC-Pd/Fe NPs. And since the free PAC molecule has been exhaustively removed by washing with water and anhydrous ethanol, the PAC molecule might chelate on Pd/Fe particle surface in the form of chemical bonds [38].

Detailed XPS survey on the region of Fe 2p of bare and cellulose-stabilized Pd/Fe NPs are presented in Fig 4(D). The Fe 2p photoelectron peaks at 710.6 eV, 718.6 eV, and 724.6 eV are assigned to Fe 2p_3/2_, Fe 2p_3/2_, and Fe 2p_1/2_, respectively. This indicates the formation of oxidized iron [25, 40]. The photoelectron peak at 706.8 eV for Fe 2p_3/2_ corresponds to Fe^0^. The area ratio of Fe^0^ in PAC-Pd/Fe, HPMC-Pd/Fe, and bare-Pd/Fe NPs curves is about 2.7:1.7:1. The increasing peak area of Fe^0^ in the spectra of cellulose-stabilized NPs suggests that the antioxidizability of NPs might be significantly enhanced when PAC and HPMC are used as stabilizers. The antioxidizability of NPs might follow the order of bare-Pd/Fe < HPMC-Pd/Fe < PAC-Pd/Fe NPs. This conclusion is in good agreement with the conclusion obtained from the XRD.

### FTIR analysis of cellulose-stabilized Pd/Fe NPs

**Fig 5:**
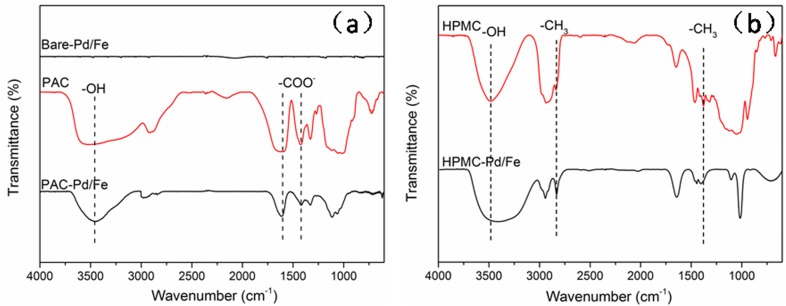
FTIR spectra of (a) PAC, bare-Pd/Fe and PAC- Pd/Fe NPs; (b) HPMC, HPMC-Pd/Fe NPs. Fe loading is 0.4 g L^-1^ with Pd 0.3 wt% of Fe, cellulose is 1 g L^-1^.

The celluloses used in this work include PAC and HPMC, in which PAC bears carboxylate and hydroxyl functional groups, while HPMC molecule only bears hydroxyl functional group, seen in Table 1. In order to unravel the stabilization mechanisms of cellulose stabilizers on Pd/Fe NPs, FTIR spectra of free celluloses, bare-Pd/Fe, and cellulose-stabilized Pd/Fe NPs were measured. The results are depicted in detail in Fig 5 and S1 Table (Supporting Information). It is generally believed that the stretching frequencies for the functional groups of celluloses are expected to shift significantly if cellulose molecules are adsorbed or bounded to the surfaces of the Pd/Fe NPs.

Fig 5(A) clearly shows that there is no apparent assignment peaks inthe bare-Pd/Fe NPs. Two apparent IR peaks are found at 1631.7 and 1419.5 cm^-1^ in FTIR spectra of PAC-Pd/Fe NPs (Fig 5(A)), corresponding to the characteristic asymmetric and symmetric carboxylate vibration, respectively [41]. For the complexation of a carboxylate group and a metal (or metallic oxide), three patterns were proposed as follows [21, 41]: (ⅰ)monodentate chelating, (ⅱ) bidentate chelating, and (ⅲ) bidentate bridging, as illustrated in S1 Fig. (Supporting Information). The separation of symmetric and asymmetric carboxylate stretching frequencies (Δ*v* = *v*_as_- *v*_s_) is used to elucidate the bonding mechanism between the carboxylate polymer and metal NPs. If 200 <Δ*v*< 320 cm^-1^, the binding is governed by monodentate interaction; if 140 <Δ*v*< 190 cm^-1^, the binding is governed by bidentate bridging interaction; if Δ*v*< 110 cm^-1^, the binding is governed by bidentate chelating interaction. In this work, the separating frequency of PAC-Pd/Fe NPs is 212.2 cm^-1^ (1631.7–1419.5 cm^-1^), which indicates that monodentate interaction is the primary binding mechanism between the PAC monomer and Pd/Fe nanoparticle surface. Similar binding mechanism has also been reported in previous studies [21, 25]. In addition, because -OH group is also present in PAC molecular, a broad peak would be obtained at 3400–3600 cm^-1^. The -OH stretching band shifted from 3510.3 cm^-1^ in free PAC to 3435.1 cm^-1^ in PAC-Pd/Fe NPs. It suggests that an intermolecular hydrogen bond could be formed between PAC molecular and nanoparticle surface, which could be regarded as the secondary binding mechanism. Therefore PAC molecules might be successfully adsorbed onto nanoparticle surface via carboxylate and hydroxyl groups in the form of chemical adsorption instead of physical adsorption.

In the case of HPMC-Pd/Fe NPs (Fig 5(B)), it could be seen that there are vibration peaks corresponding to -OH groups (3420.3 cm^-1^), C-O stretching (1021.8 cm^-1^), and -CH_3_ deformation vibrations (1408.9 cm^-1^) [33, 34]. By comparing the spectra of the free HPMC with HPMC-Pd/Fe NPs, the band corresponding to -OH stretching shifted from 3447.8 to 3373.4 cm^-1^, suggesting that -OH group of HPMC is successfully involved in forming hydrogen bond with the particle surfaces. This observation is in good agreement with the previous studies obtained by Tiwari et al. [33] and Maity et al [34].

### Stability of cellulose-stabilized Pd/Fe NP suspensions

**Fig 6:**
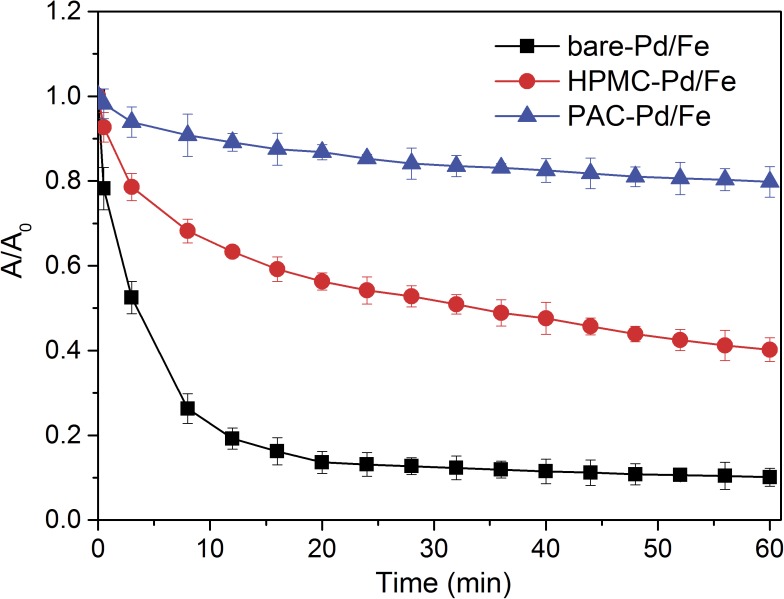
Sedimentation curves of bare and cellulose-stabilized Pd/Fe NPs. Fe concentration is 0.4 g L^-1^ with Pd 0.3 wt% of Fe, cellulose is 1 g L^-1^, respectively. Error bars represent the standard deviation of three samples.

**Fig 7:**
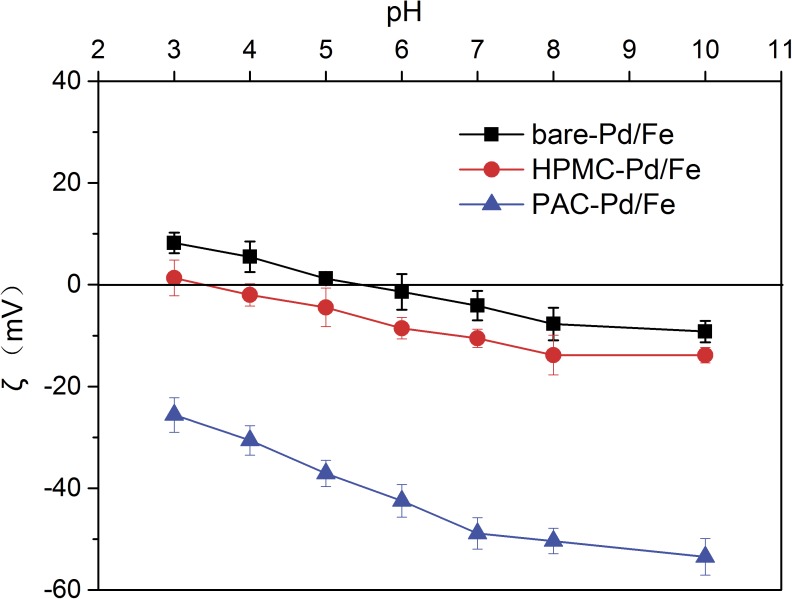
Zeta (ζ) potentials of bare-Pd/Fe, HPMC-Pd/Fe, and PAC-Pd/Fe NPs as a function of solution pH. Fe concentration is 0.4 g L^-1^ with Pd 0.3 wt% of Fe, HPMC and PAC are 1 g L^-1^, respectively. Error bars represent the standard deviation of three samples.

The stability of bare-Pd/Fe, HPMC-Pd/Fe, and PAC-Pd/Fe NPs is quantitatively evaluated by monitoring the sedimentation rates of the NP suspensions. As shown in Fig 6, the sedimentation curves clearly indicate that cellulose-stabilized Pd/Fe NPs exhibit higher stability than bare-Pd/Fe NPs, as the latter one readily settle down rapidly within 60 minutes. PAC-Pd/Fe NPs exhibit much better stability than HPMC-Pd/Fe NPs. The difference can be explicated through electrostatic repulsion and/or steric hindrance forces provided by these celluloses as stabilizers. Phenrat et al. [42] considered that the stability of NPs is governed by the sum of the interparticle interaction forces involving attraction forces (magnetic, and van der Waals forces) and repulsion forces (electrostatic, and steric forces). The ζ-potential can indicate the extent of the electrostatic interactions between NPs. In addition, literature research [43] suggested that a ζ-potential of at least ±30 mV is needed to maintain a metastable suspension. As shown in Fig 7, the ζ-potential of bare-Pd/Fe NPs solution is nearly zero, indicating that the stability of bare-Pd/Fe NPs is low. For HPMC-Pd/Fe NPs, the ζ-potential is also close to zero (seen in Fig 7). However, HPMC-Pd/Fe NPs show better stability than bare-Pd/Fe NPs, which indicates that steric hindrance plays a critical role in stabilizing Pd/Fe NPs. For PAC-Pd/Fe NPs, the addition of PAC (a anionic cellulose ether) alters the surface charge of Pd/Fe NPs such that they have a negative charge. As shown in Fig 7, the ζ-potentials of PAC-Pd/Fe NPs range from -25.6 mV to -53.5 mV. Sakulchaicharoen et al. [28] considered that enhanced NPs stability in CMC suspensions mainly resulted from electrostatic repulsion instead of steric hindrance. Therefore, we infer that the negative charges of PAC play a more important role on the stability of NPs than the molecular weight. In addition, Phenrat et al. [42] proposed that magnetic attractive forces increase with *r*^6^ (*r* is the particle radius). Thus smaller particles have significantly less magnetic attraction forces than the larger particles. As the above analysis, the particle size of PAC-Pd/Fe NPs is smaller than bare-Pd/Fe and HPMC-Pd/Fe NPs. These would explain the better suspension stability of PAC-Pd/Fe NPs than bare-Pd/Fe and HPMC-Pd/Fe NPs.

### Reactivity of cellulose-stabilized Pd/Fe NPs

**Fig 8:**
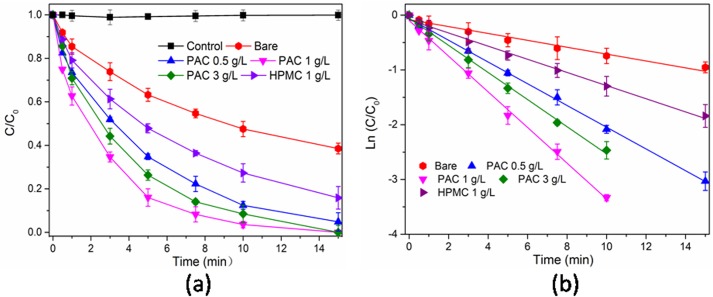
(a) Debromination of BDE47 using bare and cellulose-stabilized Pd/Fe NPs, (b) The kinetics of BDE47 debromination. Initial BDE47 concentration (*C*_*0*_) = 1 mg L^-1^, iron dose = 0.4 g L^-1^, Pd/Fe mass ratio = 0.3%, Brij35 = 1 g L^-1^. Error bars represent the standard deviation of three samples.

The debromination rates of BDE47 by bare and cellulose-stabilized Pd/Fe NPs are shown in Fig 8. Control tests show that the adsorption of BDE47 by glass flask is negligible. As shown in Fig 8(A), the debromination rates of BDE47 by bare-Pd/Fe, HPMC(1 g L^-1^)-Pd/Fe, and PAC(1 g L^-1^)-Pd/Fe NPs are 61.4%, 79.5%, and 100% within 15 minutes, respectively. This indicates that celluloses could accelerate the debromination rate of BDE47. The pseudo-first-order kinetic model was also applied to descript the debromination reactivity toward BDE47. The calculated data are shown in Table 2. All the correlation coefficients (*R*^2^) are higher than 0.96, indicating that the debromination of BDE47 followed the pseudo-first-order kinetic model. The pseudo-first-order rate constants (*k*_*obs*_, min^-1^) (Table 2) follow the order of PAC-Pd/Fe >HPMC-Pd/Fe > bare-Pd/Fe NPs. Degradation of halogenated contaminants by Fe^0^-basedparticles occurs on the surface of Fe^0^-basedparticles [11–13], therefore increasing the surface area of particles will increase the dehalogenation rate. A smaller average particle size could yield a higher surface area per mass of NPs. The mean diameter of HPMC-Pd/Fe NPs is larger than PAC-Pd/Fe NPs but smaller than bare-Pd/Fe NPs. This result is consistent with the above reactivity trend. The highest reactivity is observed when PAC is used as a stabilizer, and the result is also consistent with the size distribution of NPs.

**Table 2:** Pseudo first order kinetics analysis of BDE47 reduction using bare and cellulose-stabilized Pd/Fe NPs.

Stabilizer	*k*_*obs*_ (min^-1^)	*R*^2^	BET (m^2^g^-1^)	*k*_*SA*_ (L h^-1^ m^2^)	*t*_*1/2*_(min^-1^)
bare	0.0628	0.9687	32.3	0.2916	11.04
HPMC (1 gL^-1^)	0.1205	0.9943	43.8	0.4127	5.75
PAC (0.5 gL^-1^)	0.1984	0.9986	-	-	3.49
PAC (1 gL^-1^)	0.3255	0.9966	58.6	0.8322	2.13
PAC (3 gL^-1^)	0.2475	0.9969	-	-	2.80

The half-life period (*t*_1/2_) of BDE47 debromination (Table 2) decreases from 3.49 min to 2.13 min when the PAC concentration increases from 0.5 g L^-1^ to 1 g L^-1^. However, when PAC concentration increases further to 3 g L^-1^, the debromination rate of BDE47 is decreased. Similar results were reported by He [44], Wang [26], and Sakulchaicharoen [28] et al. Wang et al [26] studied the effect of PAA on the dechlorination rate of 2,4-dichlorophenol (2,4-DCP) by Pd/Fe NPs. They found that the dechlorination efficiency was accelerated by lower concentration of PAA, but decreased with higher concentration of PAA. The possible explanation was that excess PAA might occupy the available reactive sites of NPs and then prevent the targeted pollutants from diffusing onto the surface sites. He et al [44] also considered that excess CMC molecules could form a more compact surface coating at elevated CMC concentration, which could block available Pd/Fe NPs reactive sites and hinder the mass transfer of TCE from the bulk solution to Pd/Fe NPs reactive sites.

In addition, to better investigate the debromination performance of different Pd/Fe NPs, the values of *k*_*SA*_ (the surface area-normalized constant, L min^-1^ m^-2^) were also calculated and listed in the Table 2. The *k*_*SA*_ values of bare-Pd/Fe, HPMC-Pd/Fe, and PAC-Pd/Fe NPs reacted with BDE47 are 0.2916, 0.4127, and 0.8322 L min^-1^ m^-2^, respectively. This indicates that the debromination reactivity of three NPs follow the order of PAC-Pd/Fe > HPMC-Pd/Fe > bare-Pd/Fe NPs. It might be ascribed to the antioxidizability of cellulose coating on the NPs surface, and this result is in good agreement with the XRD and XPS analysis in this study.

## Conclusion

In this work, PAC and HPMC successfully stabilized Pd/Fe NPs via the sodium borohydride reduction method in N_2_ atmosphere. Two stabilizers (PAC and HPMC) all played important roles in control the shape and size of Pd/Fe NPs. Both cellulose-stabilized Pd/Fe NPs displayed smaller particle size, enhanced antioxidation ability, and improved suspension stability. And PAC-Pd/Fe NPs exhibited superior performance compared to HPMC-Pd/Fe NPs. PAC bearing carboxylate and hydroxyl groups was involved in interaction with NPs by polar covalent bond and hydrogen bond, while HPMC bearing only hydroxyl groups was bound to NPs by hydrogen bond. The major stabilization mechanisms for PAC-Pd/Fe NPs and HPMC-Pd/Fe NPs were electrostatic repulsion and steric repulsion, respectively. The debromination reactivity for BDE47 was the fastest for PAC-Pd/Fe NPs, followed by HPMC-Pd/Fe and bare-Pd/Fe NPs. However, excess PAC molecules inhibited the degradation rate for BDE47.

## Supporting information

S1 Fig Patterns of iron-carboxylate complexation. (TIF)

S1 Table Assignment of infrared absorption peaks for free cellulose and stabilized Pd/Fe NPs. (DOC)
