# Supplementary figures and images for: Synthesis, characterization, and debromination reactivity of cellulose-stabilized Pd/Fe nanoparticles for 2,2',4,4'-tretrabromodiphenyl ether
Source: PLoS One. 2017 Mar 29;12(3):e0174589. doi: 10.1371/journal.pone.0174589 (PMC5371346; doi:10.1371/journal.pone.0174589)

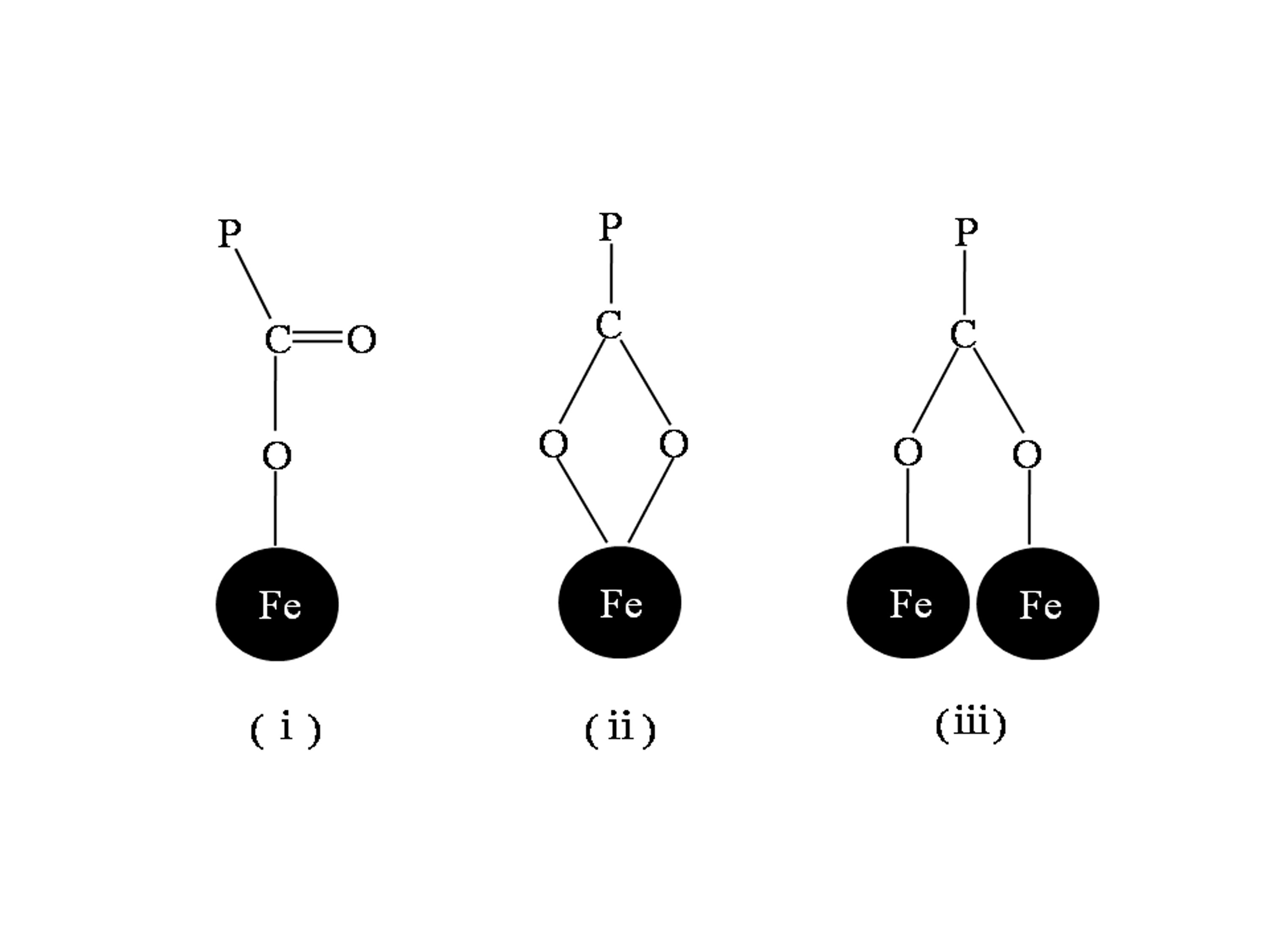

Supplement: S1 Fig — (TIF) [file pone.0174589.s001.tif]
