# Supplementary material for: Synthesis, characterization, and debromination reactivity of cellulose-stabilized Pd/Fe nanoparticles for 2,2',4,4'-tretrabromodiphenyl ether
Source: PLoS One. 2017 Mar 29;12(3):e0174589. doi: 10.1371/journal.pone.0174589 (PMC5371346; doi:10.1371/journal.pone.0174589)
Supplement: S1 Table — (DOC) [file pone.0174589.s002.doc]

**Supporting Information**

S1.Table. Assignment of infrared absorption peaks for free cellulose and stabilized Pd/Fe NPs

| Wavenumber (cm^-1^) | | Peak assignment |
| --- | --- | --- |
| PAC | PAC-Pd/Fe |  |
| 3510.3  2920.1  1604.7  1423.4  1328.9  1061.5 | 3435.1  2972.2  1631.7  1419.5  1328.9 | O-H stretching  -CH_2_ stretching  -COO^-^ asymmetric stretching  -COO^-^ symmetric stretching  -CH bending  C-O stretching |
| HPMC | HPMC-Pd/Fe |  |
| 3483.8  2935.1  2837.2  1461.3  1378.5  1113.2  1050.7 | 3420.3  2949.5  2837.2  1450.6  1408.9  1112.7  1021.8 | O-H stretching  -CH_2_ stretching  -CH_3_ stretching  -CH_2_ bending  -CH_3_ deformation  C-O-C stretching  C-O stretching |
